# Anti-Cancer Potential of Two Plasma-Activated Liquids: Implication of Long-Lived Reactive Oxygen and Nitrogen Species

**DOI:** 10.3390/cancers12030721

**Published:** 2020-03-19

**Authors:** Elena Griseti, Nofel Merbahi, Muriel Golzio

**Affiliations:** 1 CNRS UMR 5089, Institut de Pharmacologie et de Biologie Structurale, IPBS, 205 Route de Narbonne, 31077 Toulouse, France; elena.griseti@ipbs.fr; 2 CNRS UMR 5213, Laboratoire des Plasmas et Conversion d’Énergie, Université Toulouse III- Paul Sabatier, LAPLACE, 118 Route de Narbonne-Bât, 3R3-31062 Toulouse, France

**Keywords:** cancer, plasma-activated liquids, multicellular tumor spheroids, long-lived reactive oxygen and nitrogen species

## Abstract

Cold atmospheric plasma-exposed culture medium may efficiently kill cancer cells in vitro. Due to the complexity of the medium obtained after plasma exposure, less complex physiological liquids, such as saline solutions and saline buffers, are gathering momentum. Among the plethora of reactive oxygen and nitrogen species (RONS) that are produced in these plasma-activated liquids, hydrogen peroxide, nitrite and nitrate appear to be mainly responsible for cytotoxic and genotoxic effects. Here, we evaluated the anti-cancer potential of plasma-activated phosphate-buffered saline (P-A PBS) and sodium chloride 0.9% (P-A NaCl), using a three-dimensional tumor model. Two epithelial cancer cell lines were used to evaluate cellular effects of either P-A PBS or P-A NaCl. Human colorectal cancer cells HCT 116 and human ovarian carcinoma, SKOV-3 were used to investigate the manner by which different cell types respond to different plasma-activated liquids treatments. Our investigations indicate that P-A PBS is more efficient than P-A NaCl mainly because RONS are produced in larger quantities. Indeed, we show that the cytotoxicity of these liquids directly correlates with the concentration of hydrogen peroxide and nitrite. Moreover, P-A PBS induced a faster-occurring and more pronounced cell death, which arose within deeper layers of the 3D multicellular spheroid models.

## 1. Introduction

Cold atmospheric plasma (referred as ‘plasma’) is described as a room temperature ionized gas generated at atmospheric pressure. Plasma generates reactive oxygen and nitrogen species (RONS), which, once applied to cancer cells, have the potential to induce DNA damages leading to cell apoptosis [1] or cause immunogenic cell death [2,3,4]. More importantly, cancer cells appear to be more sensitive to plasma than healthy cells [5,6,7,8,9]. This selectivity could be explained by the RONS-induced stress tolerance threshold, which is higher in normal cells than cancer cells, due to their increased production of reactive oxygen species [10,11,12]. Thus, this feature could be exploited as a strategy for selective cancer therapy. As direct treatment by plasma is spatially limited to the surface, another strategy has been developed and involves the use of solutions exposed to plasma. The latter can be injected into deep-seated tissues, and could overcome the spatial limits of plasma direct applications. The exposure of cell culture media, water, or saline solutions that contain a high amount of RONS is widely studied for biomedical applications, such as regenerative medicine or cancer therapy [13,14]. Hydrogen peroxide (H_2_O_2_), nitrite (NO_2_^−^) and nitrate (NO_3_^−^) have been described as the three main RONS responsible for plasma-exposed solutions antitumor effects [15], as other short-lived species are quenched very rapidly [16]. The complex process that drives RONS diffusion/penetration within cells and the subsequent chemical reactions remain unclear. More recently, a chemical model has been proposed by Bauer, speculating that an auto-amplificatory response of tumor cells, caused by singlet oxygen, occurs after being in direct contact with plasma or with plasma-activated solutions, leading to subsequent reaction and self-perpetuation of toxicity [17]. 

Most of the studies focusing on plasma-activated culture media for cancer cell treatment show that the contents of these solutions play a key role in their anticancer effects. Indeed, the presence of amino acids, fetal bovine serum, and pyruvate have an impact on the final composition of the plasma-activated solutions and the rate of RONS produced [18,19]. Therefore, there is now a growing interest in switching to physiological and stable solutions, such as water, physiological buffers, and saline solutions, in order to have better control over the species generated after plasma exposure, with the objective of translating it to in vivo applications. A comparative study with six different physiological liquids has been reported recently and referenced their effects in vitro on 2D plated CT26 colorectal cancer cells in terms of cell death, metabolic activity, and cell morphology and displacement [20]. 

SKOV-3 and HCT-116 are both epithelial cancer cell lines. In a recent study, we demonstrated that a plasma-activated medium was efficient to kill HCT-116 cells in three-dimensional spheroid models [21]. We described the cascade of events leading to cell death: ATP depletion, DNA damages, and mitochondrial dysfunction. These effects induce cell apoptosis and spheroid volume decrease after treatment in vitro. SKOV-3 cells are known as a chemo-resistant ovarian cancer cell line. Once injected in mice, SKOV-3 disseminates and form small nodules in the peritoneal cavity. Thus, the eradication of the small aggregates formed by these cells is more challenging. Moreover, SKOV-3 are among ovarian cells, which are the most resistant to plasma treatment [22]. When treated with plasma-exposed culture medium, SKOV-3 cells were less sensitive than ES2 cells (clear cell ovarian adenocarcinoma) [23]. Using these two different cell lines pave the way for future in vivo investigations to test two different strategies of plasma-exposed solutions injection on mice (intratumoral or intraperitoneal).

In this context, we here present a comparative study involving two different physiological liquids: the phosphate-buffered saline (PBS) and the sodium chloride 0.9% solution (NaCl). In this study, we investigated the anti-cancer properties of both PBS and NaCl exposed to plasma (referred to as ‘P-A PBS’ and ‘P-A NaCl’, respectively) using a three-dimensional model, the multicellular spheroid. This model mimics an avascular micro-tumor, and is more complex than standard 2D cell cultures, enabling us to better predict in vivo response [24]. HCT 116 human colorectal cancer cell line expressing green fluorescent protein (GFP) and SKOV-3 human ovarian adenocarcinoma cell line expressing GFP and luciferase (Luc) (were used, in order to investigate the cell-line dependency on the treatment’s outcome. We evaluated the effect of P-A PBS and P-A NaCl on spheroids’ growth and correlated the treatment with the cell death kinetics. We also show that the long-lived RONS, H_2_O_2_, and NO_2_^−^ are the main factors inducing cell death within the spheroid in a dose-dependent manner. 

## 2. Results

### 2.1. Plasma-Activated PBS (P-A PBS) Affects Spheroids Growth to a Greater Extent than Plasma-Activated NaCl (P-A NaCl)

**Figure 1:**
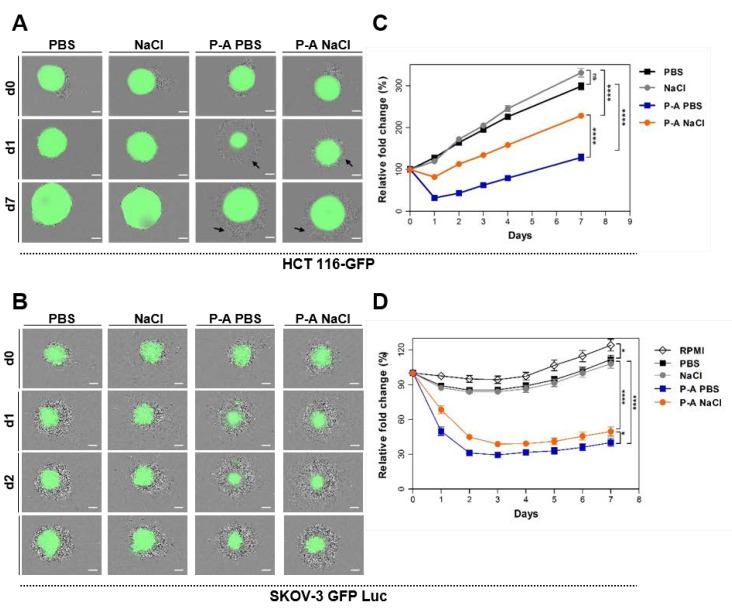
Spheroids’ responsiveness to plasma-activated PBS (P-A PBS) and plasma-activated NaCl (P-A NaCl). Spheroids were incubated in PBS, NaCl, P-A PBS or P-A NaCl for 4 h before being cultured for 7 days for growth follow-up. (**A**,**B**) Bright-field and green fluorescence micrograph overlays (viable cells) of HCT 116-GFP (**A**) and SKOV-3 GFP Luc (**B**) spheroids at d0 (before treatment), d1, d2, and d7 after treatment. The scale bar is set at 200 µm. Black arrows indicate cell debris. (**C**,**D**) Graphs representing the relative fold change (percentage) of spheroid areas as a function of time. Areas were measured from the GFP fluorescence micrographs. Two-way ANOVA, **** *p* < 0.0001; ns: non-significant *p* > 0.05. *N* = 6 and 3 independent experiments for HCT 116-GFP and SKOV-3 GFP Luc, respectively, with *n* = 8 spheroids per experiment.

We first evaluated the effect of P-A PBS and P-A NaCl on HCT 116-GFP and SKOV-3 Luc GFP spheroids’ growth (Figure 1A–D). We previously optimized the plasma exposure time and cell contact-time to 120 s and 4 h, respectively [25]. At day 5 after cells’ seeding, the spheroids underwent treatment with either P-A PBS or P-A NaCl. At the time of treatment, the spheroids exhibited different sizes, depending on the cell line (~500 µm of diameter for HCT-116 GFP and ~300 µm of diameter for SKOV-3 GFP-Luc). Figure 1A,B show bright-field and GFP fluorescence overlay micrographs. Spheroids’ growth, represented as a relative fold change in spheroid equatorial area over time, is shown in Figure 1C,D. For HCT 116-GFP spheroids, dead detached cells were observed at the external layer of the spheroids 24 h after treatment (day 1) after treatment with both plasma-activated liquids. This phenomenon reflects in growth curves, where a loss of 55% and 20% of the areas was observed 24 h after treatment with P-A PBS and P-A NaCl, respectively. During the following days, spheroids displayed a similar growth rate to the controls. After 7 days of culture, spheroids treated with P-A PBS still displayed a smaller size than spheroids treated with P-A NaCl.

A decrease of 60% and 40% of the SKOV-3 GFP Luc spheroid areas was observed one day after treatment with P-A PBS and P-A NaCl, respectively. The SKOV-3 GFP Luc spheroids appeared to be less proliferative than HCT-116 GFP as visible on growth curves of PBS and NaCl control spheroids. Thus, during the 7 days following the treatment, the spheroids growth decreased in comparison to controls. For both cell lines, spheroids responded differently to P-A PBS and P-A NaCl treatments; more precisely, P-A PBS appeared to be more efficient than P-A NaCl.

### 2.2. Plasma-Activated PBS (P-A PBS) Induces Deeper and Faster Cell Death

**Figure 2:**
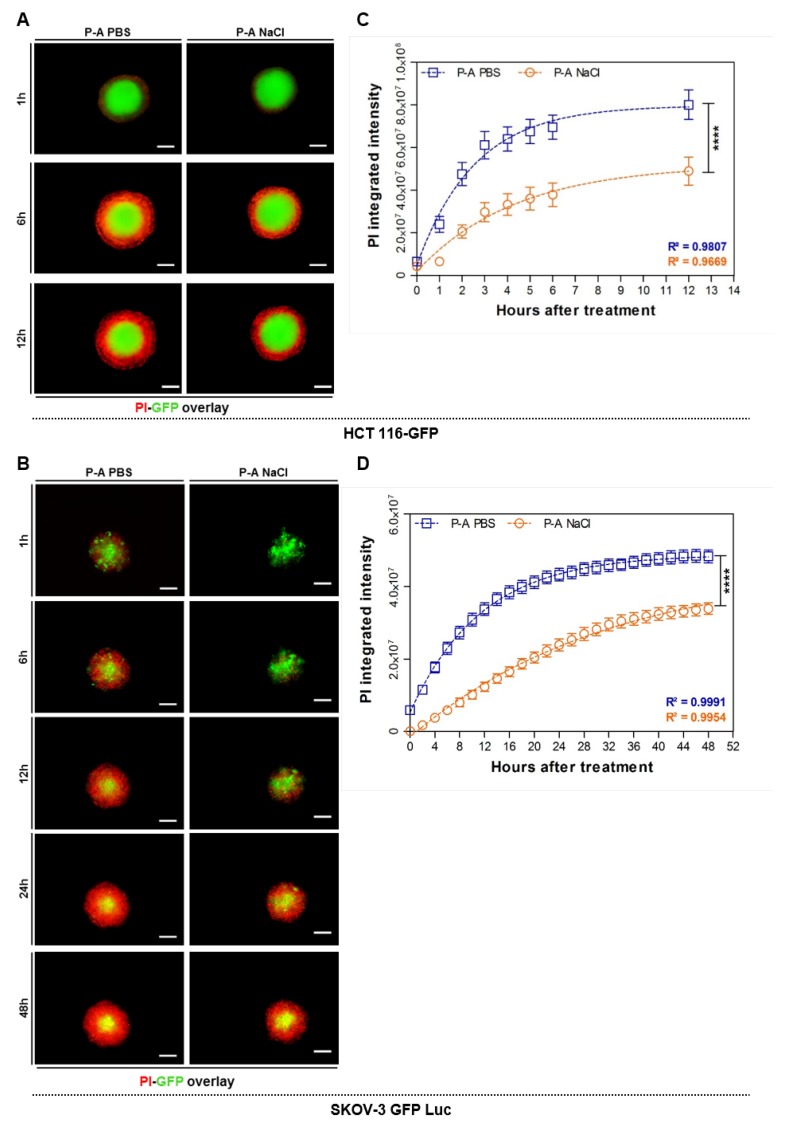
PI uptake kinetics of spheroids treated with plasma-activated PBS (P-A PBS) and plasma-activated NaCl (P-A NaCl). (**A**,**B**) Overlaid GFP and PI fluorescence micrographs of HCT-116 GFP and SKOV-3 GFP Luc spheroids, 1, 6, 12, 24, and 48 h after treatment with P-A PBS and P-A NaCl. The scale bar is set at 200 µm. (**C**,**D**) PI fluorescence integrated intensity across the spheroids as a function of time. Two-way ANOVA, **** *p* < 0.0001. Fit one-phase association equation: Y = Y_0_ + (plateau-Y_0_) × (1−e^−k*t*^). N = 3 and 2 independent experiments for HCT-116 GFP and SKOV-3 GFP Luc, respectively, with *n* = 8 spheroids per experiment.

**Figure 3:**
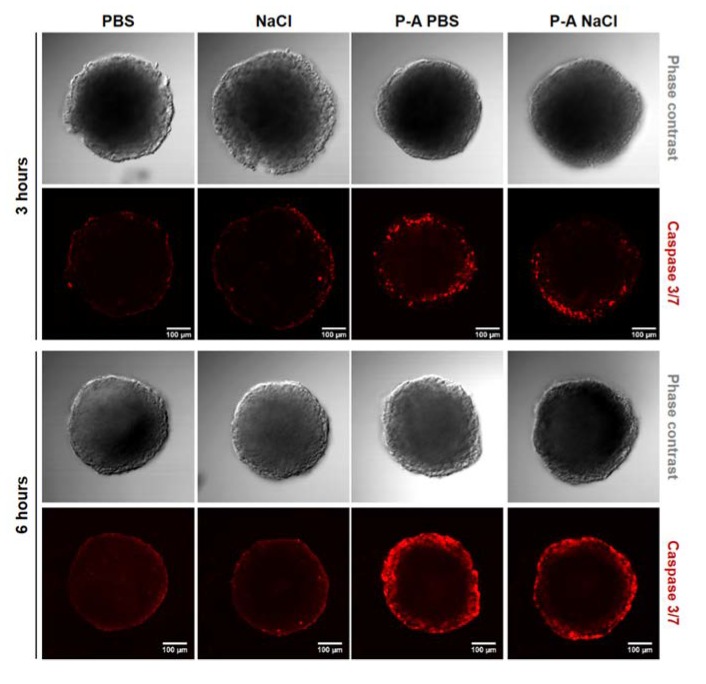
Plasma-activated liquids-induced apoptosis. HCT-116 GFP spheroids were stained with caspase 3/7 red reagent (Molecular Probes Invitrogen, Eugene, Oregon, USA)) 3 or 6 h after incubation with either PBS or NaCl exposed to plasma-activated PBS and plasma-activated NaCl (P-A PBS or P-A NaCl, respectively). Fixed entire spheroids were imaged with a confocal microscope. Phase contrast and caspase 3/7 micrographs are presented (equatorial z-slice). The emitted light from caspase 3/7 red reagent was collected through a 610–650 nm bandpass filter. PBS and NaCl represented the controls. The scale bar is set at 100 µm.

To determine the depth of cell death within the layers of multicellular spheroids, we assessed the real-time propidium iodide uptake. Propidium iodide (PI) is a red-fluorescent intercalating agent that penetrates the cells and binds to the DNA when the plasma membrane loses its integrity. PI uptake thus reflects cell mortality. As we observed that one day after treatment HCT 116-GFP spheroids were restored to a normal growth rate, we appraised the PI uptake within the 24 h following the treatment. Figure 2A shows overlaid micrographs of the PI and GFP fluorescence 1, 6, and 12 h after treatment (time-lapse video microscopy films are available in the supplementary data) on HCT 116-GFP spheroids. These micrographs confirmed that PI uptake was higher with P-A PBS treatment, compared to P-A NaCl- treated spheroids. Interestingly, these differences were visible 6 h after treatment. The PI fluorescence across the spheroid depth was plotted on an hourly basis during the first 24 h. The plots are shown in supplementary Figure S1, where each solid line corresponds to a time point (from 1 to 6, and 24 h after treatment). For both treatments, two peaks were observed at the edge of the spheroids, with the highest peak area when P-A PBS treatment was performed compared to P-A NaCl treatment. From these analyses, we set a data analysis protocol to remove the out-of-focus PI fluorescence, as described in section *H* of the “Materials and Methods” and SI (supplementary Figure S2). The kinetic of PI uptake was obtained from images (Figure 2C,D). For both cell lines, it clearly appeared that P-A PBS led to a faster penetration of PI compared to P-A NaCl. Using a non-linear regression fit, one-phase association model: Y = Y_0_ + (plateau-Y_0_) (1−e^−K*t*^), where *Y_0_* is the initial value of PI fluorescence intensity, *K* is the rate constant and *t* is time in hours, we extracted the plateau and the half times of PI uptake for each condition. For HCT 116-GFP spheroids, the plateau obtained was 1.5 times higher with P-A PBS than with P-A NaCl treatment. This indicates that the amount of PI (i.e., cell death) was more important when spheroids were treated with P-A PBS. Surprisingly, the half-time of PI uptake, corresponding to the time needed to obtain 50% of PI maximal intensity, was 1.6 times higher for P-A NaCl than for P-A PBS (P-A PBS: 1.8 h, P-A NaCl: 3 h). This increase in the P-A PBS induced mortality kinetic was also given by the rate constants (*K*) extracted from the equation. The values were 0.4 and 0.2 for P-A PBS and P-A NaCl, respectively. In accordance with the half-time, PI penetrated twice faster after P-A PBS than P-A NaCl exposure. A plateau was obtained 8 and 10 h after treatment with P-A PBS and P-A NaCl, respectively. These results were confirmed with caspases 3/7 staining on fixed entire HCT-116 GFP spheroids to compare to the apoptosis induced by the two plasma-exposed liquids (Figure 3). Three hours after treatment, few cells at the periphery were caspase 3/7 positives for P-A PBS and P-A NaCl. A concentric increase of the signal was observed in both treated conditions; however, PI-positive cells staining was more pronounced after treatment with P-A PBS. This is in accordance with the results we obtained following PI quantification.

In the case of SKOV-3 GFP Luc spheroids, a difference in the PI fluorescence profile was observed between P-A PBS and P-A NaCl treatment (Figure 2D) only one hour after the treatment. The outer shell was PI-positive for P-A PBS, whilst in spheroids treated with P-A NaCl, a longer time-lapse (8 h) was required to observe PI-positive cells at the outer rim. Non-linear regression demonstrates that the difference between the PI fluorescence profiles over time was less pronounced than in HCT 116-GFP spheroids, and the plateau obtained for P-A PBS was 1.1 times higher than for P-A NaCl. Due to a marked difference in PI fluorescence intensities during the first hours following the treatment (slope), the half-time (P-A PBS: 7.8 h, P-A NaCl: 22 h) and the rate constants K (P-A PBS: 0.08775, P-A NaCl: 0.03196) were almost 3 times higher for P-A NaCl than for P-A PBS. A plateau was obtained after 36 h and 48 h for P-A PBS and P-A NaCl, respectively.

### 2.3. Physicochemical Properties of Plasma-Activated PBS (P-A PBS) and Plasma-Activated NaCl (P-A NaCl)

**Figure 4:**
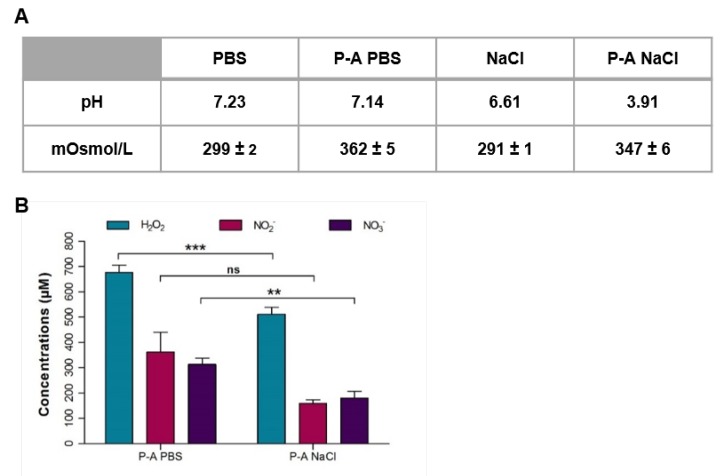
Results of the physicochemical properties analysis in plasma-activated PBS (P-A PBS) and plasma-activated NaCl (P-A NaCl). (**A**) pH and osmolarity were measured directly after exposure to the plasma jet for 120 s, as described in the Materials and Methods section I. PBS vs. P-A PBS and NaCl vs. P-A NaCl: paired *t*-test ***p < 0.001. PA-PBS vs. P-A NaCl: unpaired t-test, ns: non-significant. *N* = 3 independent experiments. (**B**) Quantification of the hydrogen peroxide (H_2_O_2_), nitrite (NO_2_^−^) and nitrate (NO_3_^−^) in P-A PBS and P-A NaCl. Unpaired t-test *** *p* < 0,001, ** *p* < 0.01; ns: non-significant *p* > 0.05. *N* = 4 independent experiments.

The only apparent difference between PBS and NaCl is the phosphate content and the buffering properties of PBS. To better understand the difference between these two plasma-activated liquids efficiency on cancer cells, we checked their physicochemical properties. We first measured the pH variation after plasma exposure. As expected, P-A PBS did not show any pH variation, and P-A NaCl displayed high acidification after exposure to plasma (from 6.61 to 3.91) (Figure 4A). When liquids were exposed to the plasma jet, 20% of the initial volume evaporated, causing variation in the osmolarity. We measured the osmolarity of the two plasma-exposed solutions. Non-exposed solutions were isotonic (300 mOsmol/L), but when exposed to plasma, a slight increase in osmolarity was observed. Variations in osmolarity were still in the range of tolerated values for cells as already reported [25]. There were no significant differences in osmolarity between P-A PBS and P-A NaCl, thus the differences between P-A PBS and P-A NaCl efficiency cannot be explained solely by the osmolarity variations.

Hydrogen peroxide (H_2_O_2_), nitrite (NO_2_^−^) and nitrate (NO_3_^−^) were referred to as the major reactive oxygen and nitrogen species responsible for plasma-activated liquids cytotoxicity [15,26]. Thus, we quantified these three species in the two plasma-activated liquids, P-A PBS and P-A NaCl (Figure 4B). Overall, a higher concentration of H_2_O_2_, NO_2_^-^ and NO_3_^−^ was produced in P-A PBS compared to P-A NaCl, H_2_O_2_ (*** *p*-value < 0.001) and NO_3_^−^ (** *p*-value <0.01) quantities were significantly different between P-A PBS and P-A NaCl. The greater anti-cancer capacity of P-A PBS could thus be explained by different concentrations observed for the three species.

### 2.4. Implication of the Hydrogen Peroxide, Nitrite and Nitrate in the Cytotoxicity of Plasma-Activated Liquids

**Figure 5:**
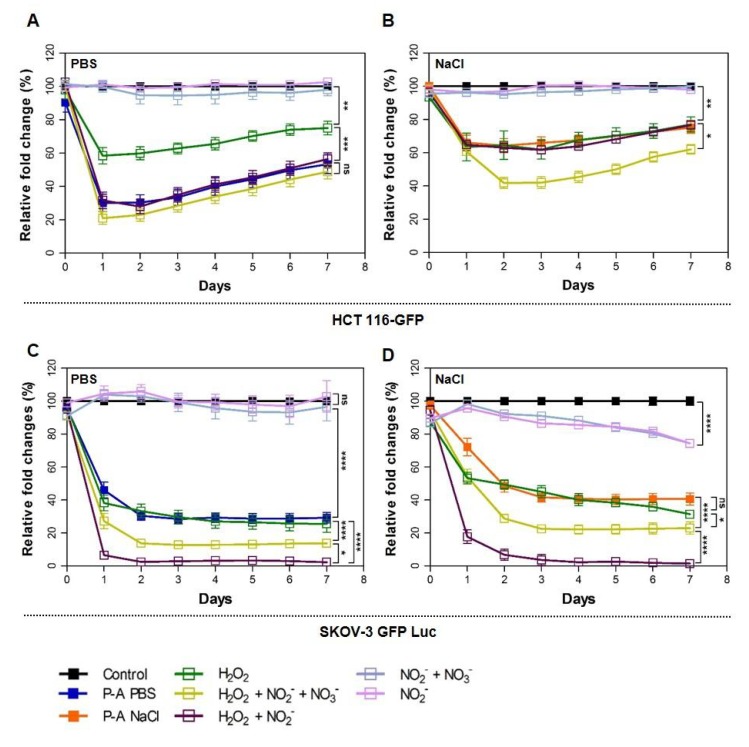
Spheroids’ treatment with long-lived reactive oxygen and nitrogen species (RONS): hydrogen peroxide (H_2_O_2_) ± nitrite (NO_2_^−^) ± nitrate (NO_3_^−^). Growth curves of spheroids incubated 4 h in PBS (control), plasma-activated PBS (P-A PBS), PBS containing 680 µM of hydrogen peroxide ± nitrite (360 µM) ± nitrate (315 µM) (**A** and **C** for HCT 116-GFP and SKOV-3 GFP Luc, respectively) or NaCl solution (control), plasma-activated NaCl **(**P-A NaCl), NaCl containing 515 µM of hydrogen peroxide ± nitrite (160 µM) ± nitrate (180 µM) (**B** and **D** for HCT 116-GFP and SKOV-3 GFP Luc, respectively). Graphs represent a relative fold change of spheroids’ equatorial area over the control in percentage as a function of time. Areas were measured from the GFP fluorescence micrographs. Untreated spheroids of HCT 116-GFP (A,B) or SKOV-3 Luc GFP (C,D) are referred to as “control”. Two-way ANOVA, **** *p* < 0.0001; *** *p* < 0.001; ** *p* < 0.01;* *p* < 0.05; *N* = 3 independent experiments with *n* = 6 spheroids per experiment for HCT 116-GFP and *N* = 2 independent experiments with *n* = 6 spheroids per experiment for SKOV-3 GFP Luc.

To understand the involvement of the three main long-lived reactive species hydrogen peroxide (H_2_O_2_), nitrite (NO_2_^−^) and nitrate (NO_3_^−^) in the cytotoxicity of the two plasma-exposed solutions, the spheroids were treated with PBS and NaCl solutions containing H_2_O_2_, NO_2_^−^ and NO_3_^−^. Based on the quantification presented above, either 680 µM of H_2_O_2_, 360 µM of NO_2_^−^, 315 µM of NO_3_^−^ or 515 µM of H_2_O_2_, 160 µM of NO_2_^−^, 180 µM of NO_3_^−^ were added to PBS and NaCl, respectively. Different combinations were used in order to point out the effect of each species. Spheroids were treated over 4 h, as with plasma-exposed solutions. The pH of the solutions was measured, and no variation was observed compared to control (PBS 7.27 and NaCl 6.86). Spheroid growth was followed during 7 days after treatment (Figure 5). For HCT 116-GFP, the treatment with PBS or NaCl containing NO_2_^−^/NO_3_^−^ or NO_2_^−^ alone did not result in differences in spheroid growth. H_2_O_2_ alone in PBS was not enough to obtain the same toxicity as P-A PBS (**** *p*-value) (showing 40% of the decrease compared to 70% obtained with P-A PBS). There were no significant differences (*p*-value > 0.05) when spheroids were treated with H_2_O_2_ + NO_2_^−^ + NO_3_^−^ and H_2_O_2_ + NO_2_^-^ without NO_3_^−^. This is in accordance with the work of Girard and co-workers [26], who showed that PBS containing H_2_O_2_ alone was not sufficient to reach the P-A PBS toxicity and acted in synergy with NO_2_^-^ to kill HCT 116 cancer cells grown in 2D cultures, and NO_3_^-^ was not needed to induce cytotoxicity.

There was no significant difference (*p*-value > 0.05) when spheroids were treated with P-A NaCl, NaCl + H_2_O_2_ and NaCl + H_2_O_2_ + NO_2_^−^ solutions. Surprisingly, NaCl solution containing H_2_O_2_ was sufficient to induce the same effect as P-A NaCl. Moreover, when both NO_2_^−^ and NO_3_^−^ were added to the solution, the latter killed cells more efficiently than P-A NaCl (* *p*-value). Precisely, the decrease in spheroids size 24 h after treatment was similar for the four conditions (P-A NaCl, NaCl + H_2_O_2_, NaCl + H_2_O_2_ + NO_2_^−^ + NO_3_^−^ and NaCl + H_2_O_2_ + NO_2_^−^). Conversely, when both NO_2_^−^ and NO_3_^−^ were present in solution, spheroid size continued to decline until day 2 after treatment.

SKOV-3 GFP Luc treated with PBS or NaCl containing H_2_O_2_ alone were affected as efficiently as P-A PBS or P-A NaCl. Surprisingly, nitrite and nitrate appeared to be toxic for SKOV-3 GFP Luc spheroids when added to NaCl solution. Moreover, a synergetic effect between the three species was observed for this cell line. Spheroids treated with the three species were more affected than spheroids treated with H_2_O_2_ and NO_2_^−^.

To check if the differences observed are RONS dose-dependent and/or due to chemical interactions between plasma and the two liquids, we treated spheroids with PBS containing the same quantity of the three species as quantified in the case of P-A NaCl and vice versa. Spheroid growth was followed (Supplementary Figure S3). We observed a dose-response phenomenon, whatever the liquid used. Cell detachment within the outer rim of treated spheroids and growth perturbation were the same for a defined quantity of RONS used. The only difference observed was that when NaCl was used, 48 h were necessary to obtain the same growth decrease as the one observed for PBS for the same RONS quantity. This is in agreement with the differences observed for PI uptake, where the kinetic of PI uptake after treatment with P-A PBS was faster than after treatment with P-A NaCl.

## 3. Discussion

Cold atmospheric plasma, and more specifically plasma-activated liquids, represent an alternative to the use of the plasma jet, which might be applied for cancer treatment. These two therapeutic strategies share a common approach: they take advantage of the RONS effect on cancer cells. Plasma-activated liquids are RONS-enriched solutions that can be generated after exposure to a plasma jet, and which remain stable for one month when stored at 4 °C. The purpose of this study was to investigate the anti-cancer properties of two different physiological saline solutions, PBS and NaCl 0.9%, exposed to plasma. These solutions differ in chemical composition and in buffering properties, and are both likely to be used for in vivo applications. Two cell lines were used to generate multicellular tumor spheroids, which produce a 3D model that closely mimics the main features of small avascular solid tumors. The HCT 116 human colorectal cancer cells form compact and highly-proliferative spheroids, while SKOV-3 cells are ovarian cancer cells derived from ascites, which are well known for their ability to form metastases and exhibit malignant tumor progenitor cells characteristics [27].

For both cell lines, the spheroids exhibited different responses to treatments with PBS and NaCl previously exposed to plasma. More precisely, the plasma-activated PBS was more efficient than plasma-activated NaCl for killing cancer cells (Figure 1), while both P-A PBS and P-A NaCl led to apoptosis in the peripheral cells of the spheroids. Thus, it would appear that cell death mediated by plasma-activated saline solutions followed the same cell death mechanism induced by the plasma-activated medium [20]. This study shows that the difference in the efficacy of two plasma-activated liquids can be directly correlated to their content in hydrogen peroxide (H_2_O_2_), nitrite (NO_2_^−^) and nitrate (NO_3_^−^) (Figure 4B). For the same plasma jet exposure time, a higher enrichment was observed in PBS in comparison to NaCl. To corroborate the implication of RONS, we also investigated the individual and combined effects of each species to better understand their involvement in cell death (Figure 5). The response to these three RONS was cell-line dependent. We showed that PBS containing H_2_O_2_ and NO_2_^-^ kill as efficiently as plasma-activated PBS. When H_2_O_2_ alone was added to PBS, limited cytotoxicity was observed. This is in accordance with the work of Girard et al. that showed that in the 2D, HCT 116 monolayer, the two species are required and act in synergy [26]. This was also confirmed by Privat-Maldonado and co-workers, who compared the effect of direct treatment with the plasma jet and indirect treatment with PBS exposed to plasma on glioblastoma spheroid models [28]. In this study, the authors highlighted the importance of short-lived species in direct treatment, but also showed that plasma-treated liquid cytotoxicity relied mainly on long-lived species. On the other hand, when added to NaCl, H_2_O_2_ alone seemed to be responsible for cell death. Moreover, there was no synergy effect with NO_2_^−^/NO_3_^−^ when they were added to the solution. Similarly, for the SKOV-3 GFP Luc spheroids, H_2_O_2_ alone, either in PBS or NaCl, was sufficient to be as effective as the plasma-activated liquids. Surprisingly, SKOV-3 GFP spheroids display higher sensitivity to PBS and NaCl containing H_2_O_2_ plus NO_2_^−^ or H_2_O_2_ plus both NO_2_^−^ and NO_3_^−^ than for plasma-activated liquids. Indeed, this result was surprising, and further studies should be performed in order to assess the reversibility of the RONS cytotoxic effect in the plasma-activated PBS and NaCl and in the PBS and NaCl added to the RONS by using quenchers such as ascorbic acid or pyruvate.

To better understand the differences observed in spheroid morphology and growth after treatment, PI penetration inside the 3D spheroids was followed over time, and non-linear regressions were extracted from these data in order to obtain the kinetics of cell death during P-A PBS and P-A NaCl treatments (Figure 2). Cells were dying more rapidly and within deeper layers when plasma-exposed PBS treatment was performed. The response to the treatment was also cell line dependent, as we observed different PI/cell death kinetics between HCT-116 GFP and SKOV-3 GFP. The SKOV-3 GFP Luc displayed a slower growth rate constant, compared to HCT 116-GFP. Precisely, the rate constant was more than 4-times higher for HCT 116-GFP compared to SKOV-3 GFP treated with both plasma-exposed saline solutions (P-A PBS and P-A NaCl), meaning that HCT 116-GFP were dying 4-times faster than SKOV-3 GFP. This sensitivity might be due to the morphological characteristics of spheroids made with different cell lines. Indeed, we observed that SKOV-3 GFP Luc cells formed smaller spheroids than HCT 116-GFP and displayed a slower growth rate. HCT 116-GFP spheroids appeared to be highly proliferative when compared to SKOV-3, which were mainly composed of quiescent cells [29].

Moreover, exposure of NaCl to the plasma jet induces acidification (Figure 4A) that of course was not observed for PBS, which is buffered. This acidic pH seems to play an important role. Chemical reactions, which lead to the production of beneficial sub-products, may have occurred in an acidic environment. It has already been described that plasma interaction with NaCl leads to nitrous acid (HNO_2_), nitric acid (HNO_3_), and hydrogen peroxide (H_2_O_2_), making the solution more acidic [30]. This acidification could also explain the differences observed in the response to P-A NaCl compared to NaCl containing the three RONS (H_2_O_2_, NO_2_^−^ and NO_3_^−^). Plasma exposure through chemical reactions with ambient air probably induces the production of other long-lived species, which leads to a lower efficiency or protection from NO_2_^−^/NO_3_^−^-induced cell stress. RONS activity or their penetration may be impaired in an acidic environment. However, the acidification, which has been considered responsible for higher cytotoxicity due to the generation of peroxynitrite [31], did not play a major role in treatments involving P-A NaCl. This result indeed requires further investigations.

## 4. Materials and Methods

### 4.1. Cell Culture

Human colorectal carcinoma cells HCT 116 (ATCC^®^ CCL-247^TM^) stably expressing green fluorescent protein (GFP) [32] were cultured in Dulbecco’s Modified Eagle Medium DMEM + 4.5 g/L of glucose (Gibco-Invitrogen, Carlsbad, CA, USA), L-Glutamine (CSTGLU00, Eurobio, Les Ulis, France) and pyruvate, supplemented with 10% of fetal bovine serum (F7524, Sigma, Saint Louis, MI, USA) and 1% of penicillin/streptomycin (P0781, Sigma, Saint Louis, MI, USA). SKOV-3 stably expressing green fluorescent protein (GFP) and luciferase (Luc) ovarian carcinoma cells from (ATCC^®^ HTB-77™) were cultured in RPMI 1640 (Eurobio Scientific), supplemented with L-Glutamine, 10% of fetal bovine serum, 1% of penicillin/streptomycin, human insulin (I9278, Sigma Aldrich, Saint-Louis, MI, USA) at 10 µg/mL and recombinant human epidermal growth factor (E9644 from Sigma Aldrich) at 20 ng/mL. Cells were kept in a humidified atmosphere at 37 °C and 5% of CO_2_ and were mycoplasma negative (as tested every week with MycoAlert Mycoplasma Detection kit, cat n°#LT07-318, Lonza, Switzerland).

### 4.2. Spheroid Formation

The non-adherent technique was used to generate spheroids. Briefly, 500 or 5000 cells (for HCT-116 GFP and SKOV-3 GFP Luc, respectively) were suspended in 200 µL of culture medium, and seeded in Costar^®^ Corning^®^ Ultra-low attachment 96 well plates (Fisher Scientific, Illkirch, France). Spheroids were kept in a humidified atmosphere at 37 °C and 5% of CO_2_. Cell aggregation occurred in the first 24 h following the seeding and allowed for obtaining single spheroids of similar sizes in each well.

### 4.3. Plasma Experimental Setup

**Figure 6:**
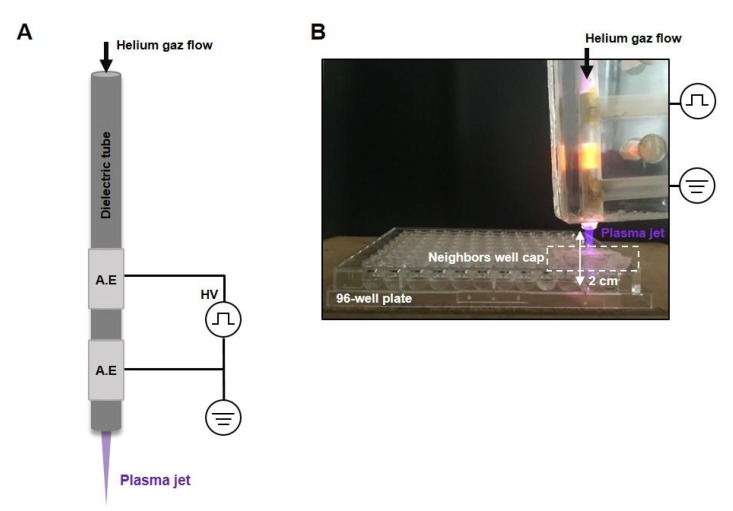
The cold-atmospheric plasma jet configuration. (**A**) Schematic representation of the plasma jet device used in our study. A.E: aluminum electrodes. (**B**) A photograph illustrating the exposure of the liquids in a 96-well plate using the plasma jet device.

A non-thermal atmospheric helium plasma jet was generated by a dielectric barrier discharge (DBD) device in ambient air as described previously [25]. The device was powered by a mono-polar square pulse of 1 µs duration with 10 kV of magnitude at 10 kHz frequency in a 4-mm inner diameter quartz dielectric tube wrapped with two 2-cm long aluminum electrodes (10 mm distance). Helium gas flow was controlled by a flow meter and fixed at 3 L/min. A schematic representation of the device and a picture of the setting for liquid activation are shown in Figure 6A.

### 4.4. Liquids Exposition

A 96-well round adherent bottom plate was used to expose the PBS (phosphate-buffered saline modified without calcium and magnesium, Eurobio, Les Ulis, France) and Sodium Chloride 0.9% injectable solution (NaCl) (Lavoisier, France). Briefly, 100 µL per well of each solution was exposed for 120 s to the plasma jet as described above, the output of the plasma jet was placed at a distance of 2 cm from the liquid surface, and a plastic cap was used to avoid interaction with neighboring wells (Figure 6B).

### 4.5. Spheroid Treatment with Plasma-Activated Liquids RONS-Containing Liquids

Spheroids were treated when their size reached 450–500 µm or 300–350 µm of diameter, for HCT-116 GFP and SKOV-3 GFP Luc, respectively (~5 days of culture). Directly after exposure to the plasma jet, a volume of 80 µL (20 µL of the solution evaporated during plasma jet exposure) of plasma-activated PBS or NaCl (P-A PBS, P-A NaCl) were transferred onto the spheroids and the plate was placed under cell culture conditions (37 °C, 5% CO_2_) for 4 hours’ incubation. When incubation time was over, the spheroids were rinsed twice with PBS, and fresh culture medium (pyruvate-free, DMEM, Gibco-Invitrogen, Carlsbad, CA, USA) was added to each well. The same protocol was followed for treatment with PBS and NaCl containing H_2_O_2_ (hydrogen peroxide solution from Sigma, France, 30% (w/w) in H_2_O and/or NO_2_^−^ and/or NO_3_^−^ (Sodium nitrite and nitrate powders from Fisher Scientific, Illkirch, France).

### 4.6. Growth Follow-Up

After treatment, the plate was placed in IncuCyte Live Cell Analysis System Microscope at ×10 magnification (Essen BioScience IncuCyte™, Herts, Welwyn Garden City, UK). Spheroid growth was followed during 7 days after treatment using bright-field and GFP channels. Micrographs exported from IncuCyte software (U.S. National Institute of Health, Bethesda, MD, USA) were analyzed with ImageJ; the area of the spheroids was determined from green-fluorescent micrographs and was plotted as relative fold change of the initial area, as a function of time.

### 4.7. Cell Death Analysis with Propidium Iodide

Propidium iodide was used to follow cell viability in real-time. The probe’s penetration within the spheroids was followed over a period of 24 h following the treatment with plasma-activated liquids. Directly after the incubation in P-A PBS and P-A NaCl, spheroids were washed with PBS as described above, and culture medium containing 1 µM of PI was added into the wells. The red fluorescence due to PI penetration was followed with the IncuCyte Live Cell Analysis System Microscope with a ×10 objective. Images were taken every hour.

### 4.8. Cleaved Caspases 3/7 Detection

Image-iT live red caspase −3 and −7 (FLICA) detection kit for microscopy (Molecular Probes Invitrogen, Eugene, OR, USA) was used according to the manufacturer’s instructions to detect caspase activation at 3 and 6 h after treatment. Directly after treatment, the spheroids were incubated in 60 µL of FLICA reagent for 45 min at 37 °C, 5% CO_2_. Subsequently, the spheroids were washed with PBS and fixed in 60 µL of kit fixative solution for 24 h at 4 °C. Fixed entire spheroids were imaged under FV1000 confocal microscope (Olympus, Rungis, France) at a magnification ×20. Emitted light from FLICA reagent was collected through a 610–650 nm bandpass filter (Texas red).

### 4.9. Image Analysis

Micrographs were analyzed using ImageJ software as described in the supplementary Figure S2. PI signals from out-of-focus planes were collected using wide-field microscopy. The out-of-focus PI fluorescence at the center of the spheroid was the result of a field depth of the microscope objective that is larger than the object diameter. In order to remove this out-of-focus fluorescence, we applied a threshold on both PI (red fluorescent) and GFP micrographs. Areas of viable spheroids were obtained from GFP fluorescence and removed from the PI micrographs. Measurements of the integrated PI fluorescence intensity were done on the final images that represented focal plane PI fluorescence.

### 4.10. Osmolality and pH Measurements

Osmolality was measured directly after the exposure of the solution to the plasma jet with a single-sample freezing point osmometer, the OSMOMAT 030 (Gonotec, Berlin, Germany) following the manufacturer’s instructions. The pH measurements were done with SevenGo Duo™ pH/conductivity meter SG23 (Mettler Toledo, Columbus, OH, USA) with freshly prepared solutions (PBS and NaCl) either exposed to plasma or supplemented with RONS).

### 4.11. Quantification of Hydrogen Peroxide

A Fluorimetric Hydrogen Peroxide Assay kit (Sigma Aldrich) was used to detect and quantify H_2_O_2_ generated in plasma-activated liquids. Directly after plasma exposure, plasma-exposed PBS/NaCl were diluted at 1/10 (detection range) and mixed with peroxidase solution and peroxidase substrate for incubation at room temperature during 30 min. Hydrogen peroxide solution (Sigma, 30% (w/w) in H_2_O) was used to obtain a calibration curve. Then, fluorescence at 590 nm was read with the plate reader CLARIOstar (BMG LABTECH, Champigny sur Marne, France)

### 4.12. Quantification of Nitrite and Nitrate

A Nitrite/Nitrate Colorimetric Assay Kit (Sigma Aldrich) was used to measure NO_2_^−^ and NO_3_^−^ in plasma-activated liquids as described by the manufacturers. First, nitrates present in the solutions were reduced to nitrites by using nitrate reductase and enzyme co-factors (2 hours’ incubation at room temperature). Then, Griess reagent was used to detect nitrites (15 min at room temperature). Absorbance at 540 nm was read with a plate reader CLARIOstar (BMG LABTECH). NaNO_2_ and NaNO_3_ kit solutions were used to obtain calibration curves.

### 4.13. Statistical Analysis

GraphPad Prism 6 software was used for statistical analyses. All quantifications were plotted as mean ± standard error mean (SEM), and overall statistical significance was set at *p*-value < 0.05. Two-way ANOVA or t-tests were performed. Linear regression analysis was used to obtain nitrite/nitrate and hydrogen peroxide calibration curves. Non-linear regression was used to analyze PI penetration kinetics.

## 5. Conclusions

Herein, we investigated the anti-cancer potential of two different plasma-activated liquids on 3-dimensional multicellular spheroids of human colorectal cancer cells and ovarian cancer cells. Our investigations indicate that PBS exposed to plasma was more efficient and penetrates deeper and faster than NaCl exposed to plasma when using a 3D cellular model. Thus, our results indicate that plasma-exposed PBS is more appropriate for the treatment of tumors in vivo. Depending on the tumor location, the solution might be injected directly into tumors (in subcutaneous HCT-116 GFP xenografts), or intraperitoneally when treating intraperitoneal malignancies (such as the ones that occur in the SKOV-3 GFP Luc tumor model). Moreover, we also show that the cytotoxicity of plasma-activated saline solutions directly correlates with the concentration of hydrogen peroxide, nitrite and nitrate, as cells responded in a dose-dependent manner when treated with these three long-lived species. The response is cell line dependent.

## Supplementary Materials

The following are available online at https://www.mdpi.com/2072-6694/12/3/721/s1, Figure S1: PI fluorescence plot profile of HCT 116-GFP spheroids, Figure S2: Scheme of the PI and GFP micrographs analysis with ImageJ software, Figure S3: Spheroids treatment with long-lived RONS: hydrogen peroxide (H_2_O_2_) ± nitrite (NO_2_^−^) ± nitrate (NO_3_^−^).
