# Supplementary material for: Anti-Cancer Potential of Two Plasma-Activated Liquids: Implication of Long-Lived Reactive Oxygen and Nitrogen Species
Source: Cancers (Basel). 2020 Mar 19;12(3):721. doi: 10.3390/cancers12030721 (PMC7140060; doi:10.3390/cancers12030721)
Supplement: Supplementary file 1 [file cancers-12-00721-s001.zip › cancers-733089-supplementary.docx]

**Supplementary Materials**

**Supplementary video 1**: Time-lapse video microscopy film showing propidium iodide real time penetration within HCT 116-GFP spheroids after P-A PBS treatment. GFP fluorescence (green) channel and PI fluorescence channel (red) are overlaid.

**Supplementary video 2**: Time-lapse video microscopy film showing propidium iodide real time penetration within HCT 116-GFP spheroids after P-A NaCl treatment. GFP fluorescence (green) channel and PI fluorescence channel (red) are overlaid.


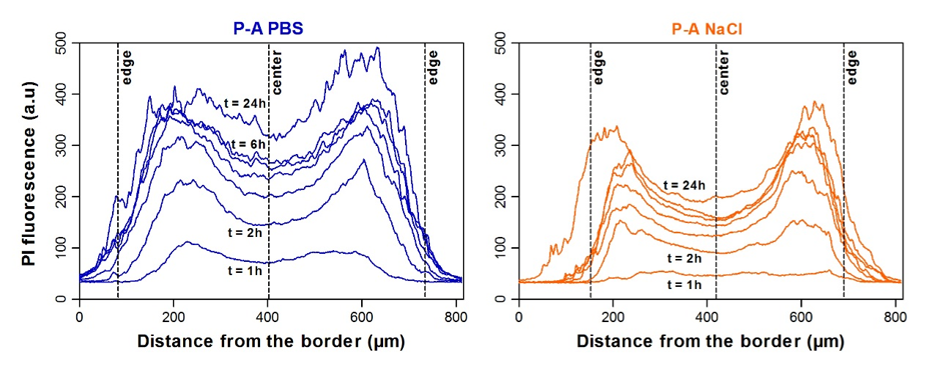


**Figure S1.** PI fluorescence plot profile of HCT 116-GFP spheroids. PI fluorescence over equatorial spheroid depth (µm) treated with plasma-activated PBS (P-A PBS), or plasma-activated NaCl (P-A NaCl) was plotted on an hourly basis during the first 24 hours. Each solid line corresponds to a time point from 1 to 6, and 24 hours after treatment.


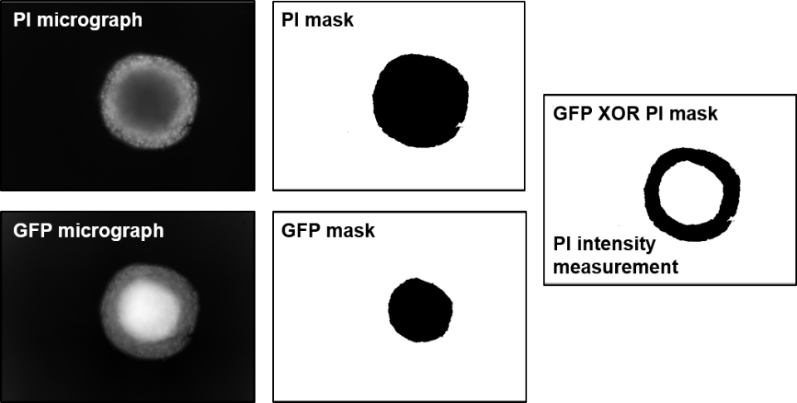


**Figure S2.** Scheme of the PI and GFP micrographs analysis with ImageJ software. PI and GFP micrographs were collected using wide field microscopy. Areas of viable spheroids were obtained from GFP fluorescence and removed from the PI micrographs. Measurements of the integrated PI fluorescence intensity was done on the final images that represented focal plane PI fluorescence.


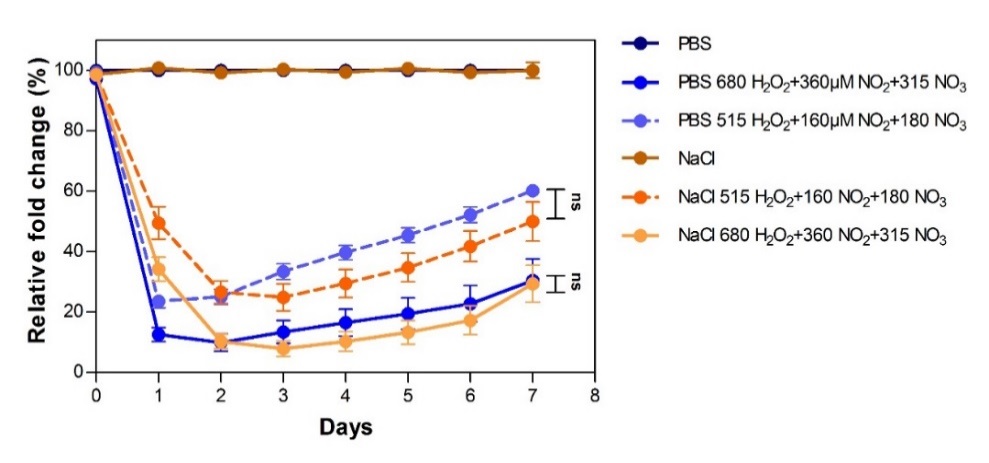


**Figure S3.** Spheroids treatment with long-lived RONS: hydrogen peroxide (H_2_O_2_) ± nitrite (NO_2_^-^) ± nitrate (NO_3_^-^). Growth curves of HCT 116-GFP spheroids incubated 4 hours in PBS (control); NaCl (control), PBS containing 680 µM of H_2_O_2_ ± NO_2_^-^ (360 µM) ± NO_3_^-^ (315 µM); NaCl containing 515 µM of H_2_O_2_ ± NO_2_^-^ (160 µM) ± NO_3_^-^ (180 µM); NaCl containing 680 µM of H_2_O_2_ ± NO_2_^-^ (360 µM) ± NO_3_^-^ (315 µM) and PBS containing 515 µM of H_2_O_2_ ± NO_2_^-^ (160 µM) ± NO_3_^-^ (180 µM). Graphs represent relative fold change of spheroids equatorial area over control in percentage as a function of time. Areas were measured from the GFP fluorescence micrographs. N=2 independent experiments with n=8 spheroids per experiment.
